# Mesenchymal stromal cells and their derivatives – putative therapeutics in the management of autoimmune pancreatitis

**DOI:** 10.1002/2211-5463.12866

**Published:** 2020-05-13

**Authors:** Robbie R. Goodman, John E. Davies

**Affiliations:** ^1^ Institute of Biomaterials and Biomedical Engineering University of Toronto Canada; ^2^ Faculty of Dentistry University of Toronto Canada; ^3^ Tissue Regeneration Therapeutics Inc Toronto Canada

**Keywords:** acute episodes, anti‐inflammatory, autoimmune pancreatitis, mesenchymal stromal cells, MSC derivatives, MSC necrobiology

## Abstract

Autoimmune pancreatitis, a derivative of chronic pancreatitis, frequently causes acute episodes with clinical symptoms parallel to those of acute pancreatitis. Corticosteroids are effective in the treatment of 90% of autoimmune pancreatitis cases, but for the remaining 10%, options are limited. Due to their significant immunomodulatory capabilities, mesenchymal stromal cells (MSCs) have been proposed as a novel treatment strategy for various immune and inflammatory pathologies including those with autoimmune origins. Here, we not only highlight the most recent MSC live‐cell experiments to address acute pancreatitis, but also discuss the opportunities afforded by the emergence of the newly identified field of MSC necrobiology. We conclude that the putative employment of MSC derivatives provides a newer and simpler therapeutic approach that could have significant advantages over the use of cells themselves.

## Graphical abstract

Autoimmune pancreatitis frequently causes acute episodes with clinical symptoms parallel to those of acute pancreatitis. Due to their significant immunomodulatory capabilities, mesenchymal stromal cells (MSCs) have been proposed as a novel treatment strategy for various immune and inflammatory pathologies including those with autoimmune origins. Here, we not only highlight the most recent MSC live‐cell experiments to address acute pancreatitis, but also discuss the opportunities afforded by the emergence of the newly identified field of MSC necrobiology.

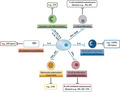

**Abbreviations**
aGVHD: acute graft‐versus‐host disease
IFN‐Υ: interferon‐Υ
IL‐1β: interleukin‐1β
MSC: mesenchymal stromal cell
OVA: ovalbumin
TNF‐α: tumor necrosis factor‐α
TSG‐6: tumor necrosis factor‐α‐induced gene/protein 6
VEGF: vascular endothelial growth factor

Autoimmune pancreatitis is a form of chronic pancreatitis that frequently causes acute episodes with clinical symptoms parallel to those of acute pancreatitis. If these acute inflammatory episodes are not properly managed, they can cause pancreatic fibrosis which can lead to the development of pancreatic cancer as well as the loss of exocrine and endocrine functions [1]. The disease is currently most commonly dealt with through the use of corticosteroids [2]. In around 90% of cases, corticosteroids are effective in the treatment of autoimmune pancreatitis [2]. However, for the other 10% of patients whose conditions do not respond favorably to corticosteroids, there is a lack of options. Of importance to note is that corticosteroids have, in some cases, been shown to provoke side effects including osteoporosis, hypertension, diabetes, weight gain, increased vulnerability to infection, cataracts, and glaucoma, thinning of the skin, and muscle weakness [3]. In terms of alternatives, the immunomodulator, thiopurine, and an anti‐CD20 monoclonal antibody, rituximab, are occasionally used to treat autoimmune pancreatitis; however, their success rates are variable [2]. For these reasons, there is considerable demand for a novel and superior strategy to manage the symptoms of autoimmune pancreatitis.

We recently (2019) summarized the available scientific literature pertaining to the putative use of mesenchymal stromal cells (MSCs) as a treatment strategy for acute pancreatitis [4]. In brief, we highlighted how MSCs, through various studies, have been proven to abrogate the effects of acute pancreatitis in rodent models. We also discussed the lack of clarity in current experimental designs that fail to take the original cause of acute pancreatitis into account. This is problematic because MSCs clearly would not be an ideal therapeutic strategy for gallstones or alcohol‐induced pancreatitis which already possesses successful treatment regimens. Here, we provide updates on new approaches reported in the field, and also discuss the use of MSC derivatives in the treatment of autoimmune pancreatitis.

Mesenchymal stromal cells were once thought to mitigate various pathologies solely through anti‐inflammatory and tissue regenerative pathways. Autologous MSC therapy gained its first regulatory approval, for the treatment of acute myocardial infarction, in 2011 [5] although not without creating some continuing controversy [6]. In fact, MSCs have been shown to exhibit an immunomodulatory phenotype through four distinct mechanisms [7] (Fig. 1). The latter include modulating the proliferation and differentiation of dendritic, B, and T cells and mediating the polarization of monocytes from an inflammatory M1 phenotype to an anti‐inflammatory M2 phenotype. In addition, MSCs can reduce the production of reactive oxygen species such as superoxide anions, which inhibit the apoptosis of neutrophils. Finally, MSCs can diminish endothelial cell responses to pro‐inflammatory cytokines such as TNF‐α, IL‐1, and IFN‐γ [7].

**Fig. 1:**
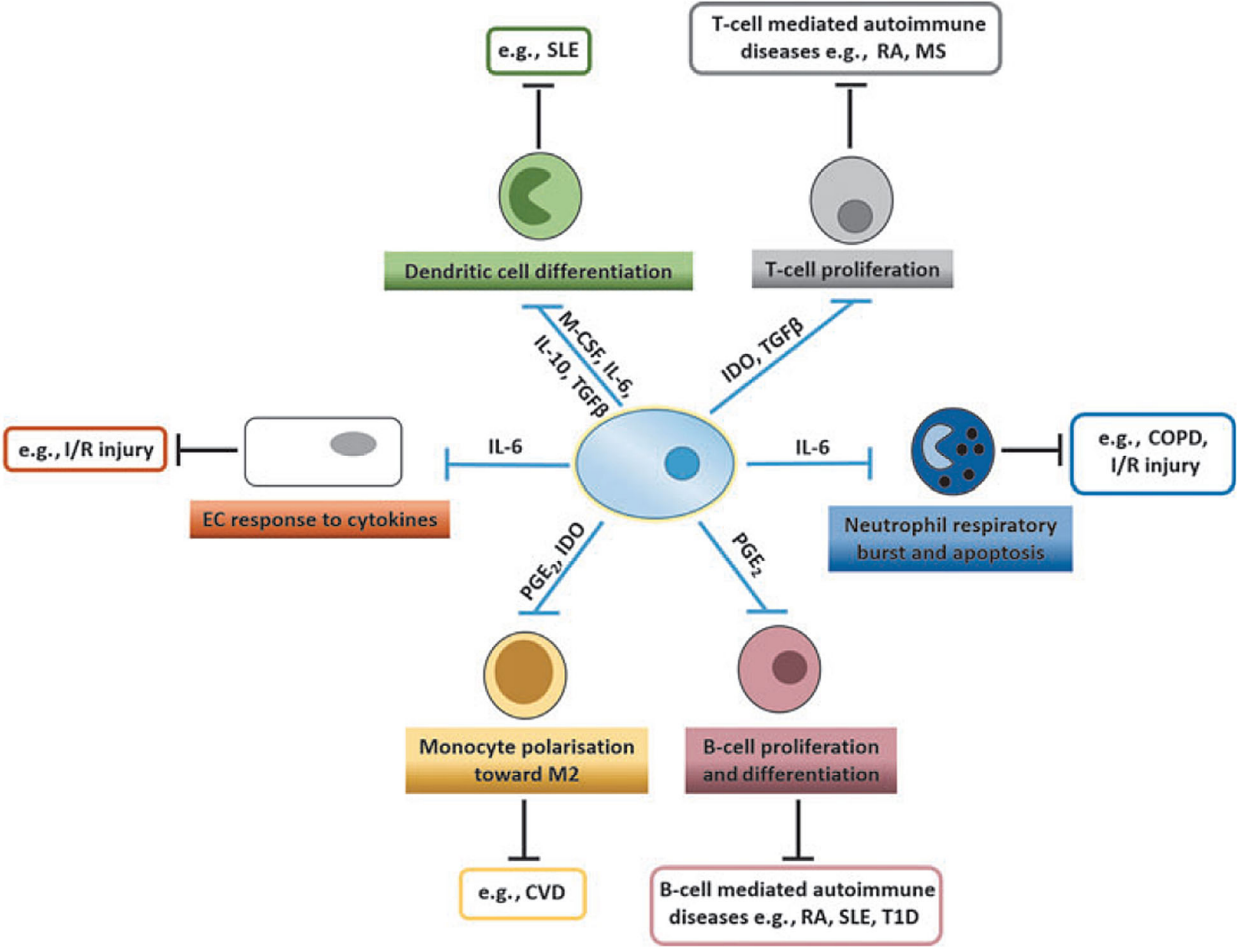
The immunomodulatory effects of MSCs all have demonstrated proven efficacy in models of chronic inflammatory and autoimmune diseases. Included with permission from Munir and McGettrick [7].

## MSCs for the treatment of immune pathologies

**Fig. 2:**
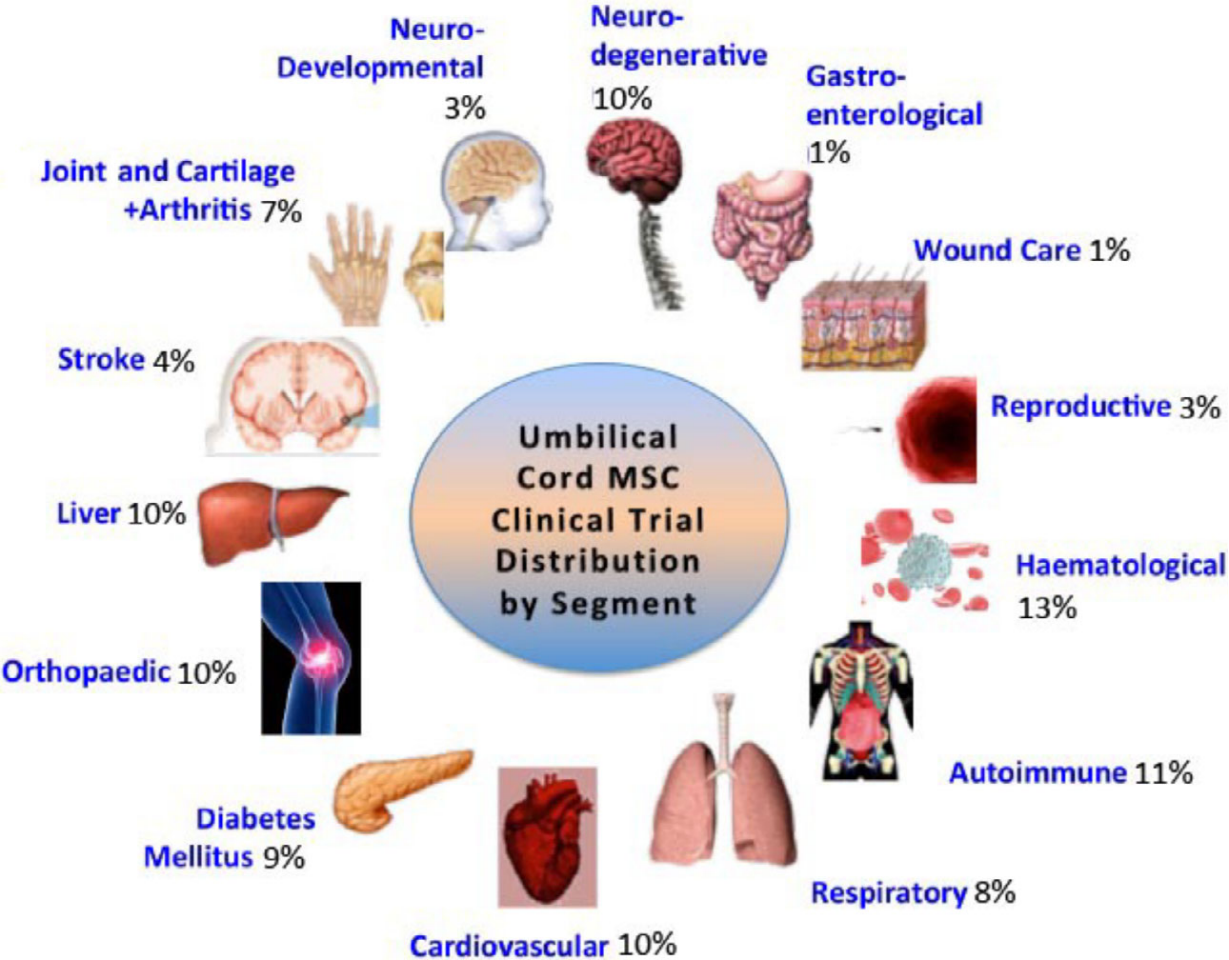
Pictorial representation of the distinct categories of pathologies for which MSC therapy is being investigated through clinical trials. Data from 954 trials were obtained by searching registered clinical trials on https://clinicaltrials.gov/, using keywords ‘mesenchymal stem cell, mesenchymal stromal cells, MSC, mesenchymal progenitor cells, multi stem cells, Pluristem PLXPAD, PDA002/001, adipose derived mesenchymal stem cell, adipose derived mesenchymal stromal cell, adipose derived MSC, ADMSC, adipose derived regenerative cell, CX610 and CX611’. Of importance to note is that this data set includes clinical trials that are recruiting, completed, or abandoned.

Due to their significant immunomodulatory capabilities, MSCs have been proposed as a novel treatment strategy for various pathologies wherein the immune system is either compromised or dysfunctional, including those with autoimmune origins (Fig. 2). Indeed, MSCs have shown success in the treatment of systemic lupus erythematosus [8], rheumatoid arthritis [9], type 1 diabetes mellitus [10], multiple sclerosis [11], liver failure associated with hepatitis B virus [12], ulcerative colitis [13], dacryoadenitis [14], Sjögren's syndrome [15], and systemic scleroderma [15]. A very recent example is the co‐administration of MSCs with pancreatic islets in immunocompetent type 1 diabetic wild‐type mice. Glycemic control was restored, using human mesenchymal cells, and a clear demonstration provided evidence of the suppression of T‐cell activation without the need for prior *ex vivo* licensing (stimulation) with the inflammatory cytokines interferon‐Υ (IFN‐Υ), interleukin‐1β (IL‐1β), and tumor necrosis factor‐α (TNF‐α) [16]. The first approval, with government reimbursement, for the treatment of an immune condition with MSCs was that in Japan in 2016, for the treatment of both pediatric disease and adult acute graft‐versus‐host disease (aGVHD) [17]. Thus, the acute inflammatory episodes of autoimmune pancreatitis may represent an appropriate therapeutic target for MSCs in cases refractory to the use of corticosteroids.

## Recent cellular approaches

One recent approach has pretreated (licensed) umbilical‐derived mesenchymal stromal cells with angiotensin II before employing them in the treatment of severe acute pancreatitis in Sprague‐Dawley rats [18]. The rationale was to maximize the constitutive angiogenic properties of UCMSCs. The pretreated cells demonstrated an enhanced ability to abrogate pancreatitis compared with cells that had not been licensed. This conclusion was established through the histological assessment of pancreatic sections using measures of necrosis, edema, vacuolization, and inflammation as well as through the observation of myeloperoxidase and serum amylase levels. The treated cells were also shown to increase the paracrine release of vascular endothelial growth factor (VEGF) which has been proven to be an important factor in pancreatic tissue healing [18].

In another study, human adipose tissue‐derived mesenchymal stromal cells were transfected with siRNA targeting tumor necrosis factor‐α‐induced gene/protein 6 (TSG‐6) and compared with their untransduced, control counterparts in the treatment of caerulein and lipopolysaccharide‐induced severe acute pancreatitis in C57BL/6 mice. Pancreas‐to‐body weight ratio, tissue edema, necrosis of acinar cells, and inflammatory cell infiltration were all improved in the control group, demonstrating the role played by TSG‐6 in ameliorating the disease. Specifically, the pro‐inflammatory cytokines, TNF‐α, IL 1β, and IL‐6 levels, and markers of endoplasmic reticulum stress, Grp78, CHOP, and caspase‐12, were decreased, while anti‐inflammatory cytokine, IL‐10, was increased in the control group [19].

## Ongoing challenges

While the above studies demonstrate convincing evidence that mesenchymal stromal cells could, in the future, be used as a novel treatment strategy for the acute episodes of autoimmune pancreatitis, it is important to acknowledge some limitations in the current scientific literature. Firstly, as we have previously discussed, to be clinically relevant, an animal model needs to be designed to specifically reflect the cause of the pancreatitis, such as autoimmune pancreatitis. Also, studies need to be conducted on the use of MSCs in large animal models whose gastroenterological anatomies are more similar to that of humans. Finally, as the cells are being proposed as a novel clinical therapy it is important to recognize barriers that could prove to be problematic as discussed below.

Mesenchymal stromal cells can be isolated from a plethora of human sources including adipose, brain, endometrial, placental, and umbilical cord tissue as well as bone marrow, cord blood, amniotic fluid, various regions of Wharton's jelly, and dental pulp [20, 21]. However, it is becoming increasingly clear that the functional phenotype of a particular population of MSCs varies with both the tissue source employed and the conditions under which the cells are culture‐expanded [22]. Despite these disparate origins, the overall abundance of MSCs in the human body is still relatively low, for example, only 0.001–0.01% of cells in the bone marrow are MSCs [20]. MSC therapy usually requires hundreds of millions of MSCs; in the aforementioned studies, 1 million cells were used in the study conducted on mice and 10 million cells were used on the rats [18, 19]. In an ongoing Phase III, human clinical trial being conducted for chronic graft‐versus‐host disease, a dose of 2 million cells per kilogram was injected six times intravenously [23]. Clearly, due to the scarce number of MSCs in the human body and the large number needed for putative therapy, a barrier might exist wherein the demand for cells needed for therapy cannot be met. *In vitro* cell expansion is the only way to yield such a high volume of cells, but this can take up to 10 weeks [24]. In addition, as described in the aforementioned studies, transfected or pretreated (licensed) cells have been proposed, which are more complicated and costly to produce.

## Live cells, dead cells, and derivatives

Therapy with live MSCs has a singular advantage over the use of their derivatives: The cells can differentiate to provide the connective tissue stroma of the recipient organ [25]. However, although MSCs have been proposed to assist with the significant stromal tissue damage that occurs in pancreatitis, the cell implantation time is too short for differentiation to occur. The two most common methods of MSC delivery for acute pancreatitis are intravenous and intraperitoneal. Less than 1% of MSCs survive for over a week after intravenous administration [26, 27], while when injected intraperitoneally, after 20 min MSCs fail to be detected in peritoneal lavage fluid and begin to form large aggregates [28]. This evidence suggests that the main therapeutic benefit of mesenchymal stromal cells is in their immunomodulatory capacity. Thus, the therapeutic advantage of live MSCs over derivatives does not extend to the cells' immunoregulatory properties.

Recent literature describes the new and exciting field of ‘MSC necrobiology’, which proposes a novel solution to some of the aforementioned barriers. Necrobiology provides four mechanisms by which derivatives of mesenchymal stromal cells can maintain significant clinical efficacy: apoptosis, autophagy, mitochondrial transfer, and extracellular vesicles [29]. MSC products derived from these pathways have been proven to ameliorate a plethora of pathologies (Table 1). Indeed, MSC derivatives (Fig. 2) have, in some cases, been shown to be as therapeutically beneficial as living MSCs [30, 31, 32, 33], but have the advantage of being able to pass easily through the circulatory system.

**Table 1:** Details of published studies investigating the therapeutic efficacy of MSC derivatives originating from four distinct necrobiology pathways.

Authors	Mechanism/Pathway	Condition(s) of interest	Results
Galleu *et al*. [34]	Apoptosis	Allergic airway inflammation and Graft‐versus‐host disease	↓ Eosinophil infiltrate in bronchoalveolar lavage (BAL) and ↓ Inflammatory endpoints ↑ Immunomodulation
Chang *et al*. [35]	Apoptosis	Sepsis syndrome induced by cecal puncture and ligation	↓ Cecal and kidney injury ↓ TNF‐α
Chen *et al*. [36]	Apoptosis	Kidney injury induced by sepsis	↓ Kidney injury
Sung *et al*. [37]	Apoptosis	Lung and kidney injury induced by sepsis syndrome	↓ Lung and kidney injury ↓ TNF‐α ↓ NF‐κB
Gao *et al*. [38]	Autophagy	This study investigated generally if the activation of autophagy in MSCs ameliorates their immunosuppressive capacity	↑ TGF‐β1 secretion ↑ Immunomodulation
Yao *et al*. [39]	Mitochondrial transfer	Asthma	↓ Asthma inflammation ↓ IL‐4, IL‐5, IL‐13
Li *et al*. [40]	Mitochondrial transfer	Airway epithelial cell damage induced by cigarette smoke in chronic obstructive pulmonary disorder (COPD)	↓ Lung injury
Zhang *et al*. [41]	Mitochondrial transfer	Cardiomyopathy	Improved heart function
Morrison *et al*. [42]	Mitochondrial transfer	Acute respiratory distress syndrome (ARDS)	↑ M2 macrophage expression ↓ TNF‐α ↓ IL‐8
Collino *et al*. [43]	Extracellular vesicle derivation	Acute kidney injury	↑ Renal tubular cell proliferation ↓ Renal tubular cell apoptosis
Lai *et al*. [44]	Extracellular vesicle derivation	Myocardial ischemia/reperfusion injury	↑ Cardioprotective effects
Li *et al*. [45]	Extracellular vesicle derivation	Acute lung injury	↓ Oxidative stress injury ↓ Inflammatory response
Wu *et al*. [46]	Extracellular vesicle derivation	Cutaneous injury	↑ Wnt4 ↑14‐3‐3ζ protein ↑ Wound healing

Mesenchymal stromal cells that have undergone apoptosis have been shown to reduce inflammatory endpoints in mouse models of allergic airway inflammation induced by ovalbumin (OVA) causing noncytotoxic T helper‐type cell inflammation [34]. Apoptotic rat adipose‐derived MSCs have also been proven superior in ameliorating the condition of cecal ligation and puncture‐induced sepsis in rat models in comparison with living MSCs [35, 36, 37]. Autophagic mesenchymal stromal cells have also been proven to have therapeutic benefits. MSCs derived from human bone marrow that have undergone autophagy have been proven to regulate CD4+ T helper cells via TGF‐β1 signaling [38]. Similarly, when MSCs underwent autophagy as a result of the administration of rapamycin, their ability to suppress CD4+ T helper cell proliferation was improved [38]. MSCs subjected to mitochondrial transfer also seem to be effective in mitigating the symptoms of asthma [39], chronic obstructive pulmonary disorder [40], cardiomyopathy [41], acute respiratory distress syndrome, and sepsis [42]. There is also evidence that extracellular vesicles derived from MSCs hold clinical efficacy in pathologies of the kidney [43], heart [44], lung [45], and skin [46].

In recent years, exosome therapy for certain inflammatory conditions has garnered significant interest. Almost all cells in the human body can produce exosomes: Extracellular vesicles produced by an endosomal pathway are approximately 30–150 nm in size [47]. Exosomes were previously thought of as having the sole responsibility of clearing cellular debris; however, recent scientific literature points to their ability to modulate intracellular environmental conditions. It is believed that only certain exosomes are immunoregulatory. Thus, a subclass of exosomes has been described as ‘signalosomes’, which, when released from MSCs, have an exceptional capacity for immunoregulation [47].

Exosome therapy using ‘signalosomes’ has been investigated for a plethora of pathologies including diseases of the heart [48], lung [49], kidney [50], brain [51], liver [52], intestines [53], skin [54], and nervous system [55] as well as for sepsis [56] and various cancers [57, 58]. In 2014, a clinical trial was conducted using exosome therapy for steroid‐refractory graft‐vs‐host disease and showed positive results [59]. Another study that investigated the use of exosomes as a novel therapeutic strategy for neonatal chronic lung disease compared their efficacy to that of a whole MSC population [60]. The study indicated that exosomes themselves hold an equivalent and sometimes even superior ability to modulate the inflammatory response. The use of MSC derivatives for treatment holds several advantages over the use of a live MSC population. They can be less costly to produce and can last up to 6 months when stored at −20 °C [61]. Derivatives are also a safer treatment option compared with whole MSCs as there are no risks associated with mutagens or oncogenicity. However, it should be pointed out that there is no documented technique to isolate ‘signalosomes’ from exosomes derived from other biogenic pathways, which may not possess the same immunomodulatory capacity [47].

Exosomes, in general, can be isolated by several methods. One of the most common methods is ultracentrifugation, although this requires costly apparatus (for industrial scale‐up) and the technique can promote vesicle aggregation which is detrimental to the structural and metabolic integrity of the exosomes produced [62]. Milder techniques of isolation have been described, for example, gradient density isolation and size‐exclusion chromatography. However, these techniques also have issues as density‐based separation may lead to enrichment as opposed to true isolation which may result in the presence of unwanted particles [63, 64]. Additionally, this technique does not allow for the large‐scale bioprocessing of exosomes, therefore making it an unrealistic technique in the context of therapy [63, 64].

Fortunately, there are other MSC‐derived products that are disruptive since production technology is far simpler and less costly than producing exosomes. Studies have been conducted indicating that cellular lysates have a similar therapeutic benefit in the treatment of various pathologies as compared to exosomes and whole‐cell MSCs themselves. Eleven published studies use cellular lysate derived from mesenchymal stromal cells from different origins to treat diverse conditions. The studies investigate the lysate's effect on aging [65], erectile dysfunction [66], fulminant hepatic failure [67], stroke [68], osteosarcoma and mammary carcinoma [69], epilepsy [70], liver failure [71], wound healing [72], ischemia [73], lymphoma [74], and obesity [75]. All of the aforementioned studies except the study investigating its effects on aging demonstrated that the lysate was able to ameliorate *in vitro* and *in vivo* models.

Recently, MSC derivative therapy has been proven effective in treating pathologies that are autoimmune in nature. Apoptotic MSCs have demonstrated immunosuppressive properties in mouse models of GVHD [38], and extracellular vesicles have improved the symptoms associated with uveitis/uveoretinitis and type I diabetes mellitus [76]. In addition, exosomes have shown success in ameliorating autoimmune conditions including multiple sclerosis [77], Sjögren's syndrome [78], graft versus host disease [59], systemic lupus erythematosus [79], and rheumatoid arthritis [80]. These results suggest the potential use of derivatives for other autoimmune conditions that lack entirely favorable treatment regimens, such as autoimmune pancreatitis.

## Conclusion

In conclusion, employing MSCs in the treatment of autoimmune pancreatitis remains a promising putative cell therapy. However, the recent emergence of a variety of methods to produce MSC derivatives provides a newer and simpler approach that could have significant advantages over the use of cells themselves including simpler production, lower regulatory barriers, and easier systemic transport upon intravenous delivery.

## Conflict of interest

The authors declare no conflict of interest.

## References

[1] Guda N and Nøjgaard C (2015) Recurrent acute pancreatitis and progression to chronic pancreatitis. Pancreapedia: The Exocrine Pancreas Knowledge Base 47, 653–666.

[2] Okazaki K , Uchida K , Sumimoto K , Mitsuyama T , Ikeura T and Takaoka M (2014) Autoimmune pancreatitis: pathogenesis, latest developments and clinical guidance. Ther Adv Chronic Dis 5, 104–111. 24790726 10.1177/2040622314527120 PMC3992826

[3] Buchman AL (2001) Side effects of corticosteroid therapy. J Clin Gastroenterol 33, 289–294. 11588541 10.1097/00004836-200110000-00006

[4] Goodman RR , Jong MK and Davies JE (2019) Concise review: The challenges and opportunities of employing mesenchymal stromal cells in the treatment of acute pancreatitis. Biotechnol Adv, 30005–30005. 10.1016/j.biotechadv.2019.01.005 30639517

[5] Yang H (2011) South Korea's stem cell approval. Nat Biotechnol 29, 857.

[6] Sipp D , Robey PG and Turner L (2018) Clear up this stem‐cell mess. Nature 561, 455–457. 30258150 10.1038/d41586-018-06756-9

[7] Munir H and McGettrick HM (2015) Mesenchymal stem cell therapy for autoimmune disease: risks and rewards. Stem Cells and Dev 24, 2091–2099. 10.1089/scd.2015.0008 26068030

[8] Wang D , Zhang H , Liang J , Wang H , Hua B , Feng X , Gilkeson GS , Farge D , Shi S and Sun L (2018) A long‐term follow‐up study of allogeneic mesenchymal stem/stromal cell transplantation in patients with drug‐resistant systemic lupus erythematosus. Stem Cell Reports 10, 933–941. 29478901 10.1016/j.stemcr.2018.01.029 PMC5918528

[9] Wang L , Huang S , Li S , Li M , Shi J , Bai W , Wang Q , Zheng L and Liu Y (2019) Efficacy and safety of umbilical cord mesenchymal stem cell therapy for rheumatoid arthritis patients: a prospective phase I/II study. Drug Des Devel Ther 13, 4331–4340. 10.2147/DDDT.S225613 PMC6930836 31908418

[10] Wu H and Mahato RI (2014) Mesenchymal stem cell‐based therapy for type 1 diabetes. Discov Med 17, 139–143. 24641956

[11] Dulamea A (2015) Mesenchymal stem cells in multiple sclerosis – translation to clinical trials. J Med Life 8, 24–27. PMC4397514 25914733

[12] Shi M , Zhang Z , Xu R , Lin H , Fu J , Zou Z , Zhang A , Shi J , Chen L , Lv S *et al* (2012) Human mesenchymal stem cell transfusion is safe and improves liver function in acute‐on‐chronic liver failure patients. Stem Cells Transl Med 1, 725–731. 23197664 10.5966/sctm.2012-0034 PMC3659658

[13] Hu J , Zhao G , Zhang L , Qiao C , Di A , Gao H and Xu H (2016) Safety and therapeutic effect of mesenchymal stem cell infusion on moderate to severe ulcerative colitis. Exp Ther Med 12, 2983–2989. 27882104 10.3892/etm.2016.3724 PMC5103734

[14] Lu X , Wang X , Nian H , Yang D and Wei R (2017) Mesenchymal stem cells for treating autoimmune dacryoadenitis. Stem Cell ResTher 8, 126. 10.1186/s13287-017-0593-3 PMC5460436 28583168

[15] Sánchez‐Berná I , Santiago‐Díaz C and Jiménez‐Alonso J (2015) Immunomodulatory properties of stem mesenchymal cells in autoimmune diseases. Med Clin 144, 88–91. 10.1016/j.medcli.2014.01.014 24636281

[16] Forbes S , Bond AR , Thirlwell KL , Burgoyne P , Samuel K , Noble J , Borthwick G , Colligan D , McGowan NWA , Lewis PS *et al* (2020) Human umbilical cord perivascular cells improve human pancreatic islet transplant function by increasing vascularization. Sci Transl Med 12, 5907. 10.1126/scitranslmed.aan5907 31941825

[17] https://www.globenewswire.com/news-release/2016/02/24/813541/0/en/First-Allogeneic-Cell-Therapy-Product-Launched-in-Japan-by-Mesoblast-Licensee.html.

[18] Yang J , Su J , Xi S , Ke X , Zhu Y , Lin H , Zeng X , Liu B , Zhu M , Dai WY *et al* (2019) Human umbilical cord mesenchymal stem cells pretreated with angiotensin‐II attenuate pancreas injury of rats with severe acute pancreatitis. Biomed Pharmaco 117, 109052. 10.1016/j.biopha.2019.109052 31176170

[19] Li Q , Song W , Ryu M , Nam A , An J , Ahn J , Bhang DH , Jung YC and Youn H (2018) TSG‐6 secreted by human adipose tissue‐derived mesenchymal stem cells ameliorates severe acute pancreatitis via ER stress downregulation in mice. Stem Cell Res Ther 9, 255–255. 30257717 10.1186/s13287-018-1009-8 PMC6158864

[20] Marquez‐Curtis L , Janowska‐Wieczorek A , Mcgann LE and Elliott JAW (2015) Mesenchymal stromal cells derived from various tissues: biological, clinical and cryopreservation aspects. Cryobiology 71, 181–197. 26186998 10.1016/j.cryobiol.2015.07.003

[21] Davies JE , Walker JT and Keating A (2017) Concise review: Wharton's jelly: the rich, but enigmatic, source of mesenchymal stromal cells. Stem Cells Transl Med 6, 1620–1630. 28488282 10.1002/sctm.16-0492 PMC5689772

[22] Boland LK , Burand AJ , Boyt DT , Dobroski H , Di L , Liszewski JN , Schrodt MV , Frazer MK , Santillan DA and Ankrum JA (2019) Nature vs. nurture: defining the effects of mesenchymal stromal cell isolation and culture conditions on resiliency to palmitate challenge. Front Immunol. 10, 1080. 31134100 10.3389/fimmu.2019.01080 PMC6523025

[23] https://clinicaltrials.gov/ct2/show/NCT02291770.

[24] Vizoso F , Eiro N , Cid S , Schneider J and Perez‐Fernandez R (2017) Mesenchymal stem cell secretome: toward cell‐free therapeutic strategies in regenerative medicine. Int J Mol Sci 18, 1852. 10.3390/ijms18091852 PMC5618501 28841158

[25] Hwang NS , Zhang C , Hwang Y and Varghese S (2009) Mesenchymal stem cell differentiation and roles in regenerative medicine. Wiley Interdiscip Rev Syst Biol Med 1, 97–106. 20835984 10.1002/wsbm.26

[26] Eggenhofer E , Benseler V , Kroemer A , Popp FC , Geissler EK , Schlitt HJ , Baan CC , Dahlke MH and Hoogduijn MJ (2012) Mesenchymal stem cells are short‐lived and do not migrate beyond the lungs after intravenous infusion. Front Immunol 3, 297–297. 23056000 10.3389/fimmu.2012.00297 PMC3458305

[27] Lee RH , Pulin AA , Seo MJ , Kota DJ , Ylostalo J , Larson BL , Semprun‐Prieto L , Delafontaine P and Prockop DJ (2009) Intravenous hMSCs improve myocardial infarction in mice because cells embolized in lung are activated to secrete the anti‐inflammatory protein TSG‐6. Cell Stem Cell 5, 54–63. 19570514 10.1016/j.stem.2009.05.003 PMC4154377

[28] Bazhanov N , Ylostalo J , Bartosh T , Tiblow A , Mohammadipoor A , Foskett A and Prockop D (2016) Intraperitoneally infused human mesenchymal stem cells form aggregates with mouse immune cells and attach to peritoneal organs. Stem Cell Res Ther 7, 27. 26864573 10.1186/s13287-016-0284-5 PMC4748482

[29] Weiss DJ , English K , Krasnodembskaya A , Isaza‐Correa J , Hawthorne IJ and Mahon BP (2019) The necrobiology of mesenchymal stromal cells affects therapeutic efficacy. Front Immunol 10, 1228. 31214185 10.3389/fimmu.2019.01228 PMC6557974

[30] Bakhtyar N , Jeschke M , Herer E , Sheikholeslam M and Amini‐Nik S (2018) Exosomes from acellular Wharton's jelly of the human umbilical cord promotes skin wound healing. Stem Cell Res Ther 9, 193. 30005703 10.1186/s13287-018-0921-2 PMC6044104

[31] Gonçalves F , Luk F , Korevaar S , Bouzid R , Paz A , López‐Iglesias C , Baan C , Merino A and Hoogduijn M (2017) Membrane particles generated from mesenchymal stromal cells modulate immune responses by selective targeting of pro‐inflammatory monocytes. Sci Rep 7, 12100. 28935974 10.1038/s41598-017-12121-z PMC5608915

[32] van Koppen A , Joles JA , van Balkom Bas W M , Lim SK , de Kleijn D , Giles RH , Verhaar MC and Dussaule J (2012) Human embryonic mesenchymal stem cell‐derived conditioned medium rescues kidney function in rats with established chronic kidney disease. PLoS ONE 7, e38746. 22723882 10.1371/journal.pone.0038746 PMC3378606

[33] Timmers L , Lim SK , Arslan F , Armstrong JS , Hoefer IE , Doevendans PA , Piek JJ , El Oakley RM , Choo A , Lee CN *et al* (2008) Reduction of myocardial infarct size by human mesenchymal stem cell conditioned medium. Stem Cell Res 1, 129–137. 10.1016/j.scr.2008.02.002 19383393

[34] Galleu A , Riffo‐Vasquez Y , Trento C , Lomas C , Dolcetti L , Cheung TS , Von Bonin M , Barbieri L , Halai K , Ward S *et al* (2017) Apoptosis in mesenchymal stromal cells induces in vivo recipient‐mediated immunomodulation. Sci Transl Med 9, 7828–7839. 10.1126/scitranslmed.aam7828 29141887

[35] Chang C , Leu S , Sung H , Zhen Y , Cho C , Chen A , Tsai T , Chung S , Chai H , Sun CK *et al* (2012) Impact of apoptotic adipose‐derived mesenchymal stem cells on attenuating organ damage and reducing mortality in rat sepsis syndrome induced by cecal puncture and ligation. J Transl Med 10, 244–244. 23217183 10.1186/1479-5876-10-244 PMC3543276

[36] Chen H , Lin K , Wallace CG , Chen Y , Yang C , Leu S , Chen Y , Sun C , Tsai T , Chen YL *et al* (2014) Additional benefit of combined therapy with melatonin and apoptotic adipose‐derived mesenchymal stem cell against sepsis‐induced kidney injury. J Pineal Res 57, 16–32. 24761983 10.1111/jpi.12140

[37] Sung P , Chang C , Tsai T , Chang L , Leu S , Chen Y , Yang C , Chua S , Yeh K , Chai HT *et al* (2013) Apoptotic adipose‐derived mesenchymal stem cell therapy protects against lung and kidney injury in sepsis syndrome caused by cecal ligation puncture in rats. Stem Cell Res Ther 4, 155–155. 24451364 10.1186/scrt385 PMC4054966

[38] Gao L , Cen S , Wang P , Xie Z , Liu Z , Deng W , Su H , Wu X , Wang S , Li J *et al* (2016) Autophagy improves the immunosuppression of CD4+ T cells by mesenchymal stem cells through transforming growth factor‐β1. Stem Cells Transl Med 5, 1496–1505. 27400793 10.5966/sctm.2015-0420 PMC5070508

[39] Yao Y , Fan X , Jiang D , Zhang Y , Li X , Xu Z , Fang S , Chiu S , Tse H , Lian Q *et al* (2018) Connexin 43‐mediated mitochondrial transfer of iPSC‐MSCs alleviates asthma inflammation. Stem Cell Reports 11, 1120–1135. 30344008 10.1016/j.stemcr.2018.09.012 PMC6234920

[40] Li X , Zhang Y , Yeung SC , Liang Y , Liang X , Ding Y , Ip MSM , Tse H , Mak JCW and Lian Q (2014) Mitochondrial transfer of induced pluripotent stem cell‐derived mesenchymal stem cells to airway epithelial cells attenuates cigarette smoke‐induced damage. Am J Respir Cell Mol Biol 51, 455–465. 24738760 10.1165/rcmb.2013-0529OC

[41] Zhang Y , Yu Z , Jiang D , Liang X , Liao S , Zhang Z , Yue W , Li X , Chiu S‐M , Chai Y‐H *et al* (2016) iPSC‐MSCs with high intrinsic MIRO1 and sensitivity to TNF‐a yield efficacious mitochondrial transfer to rescue anthracycline‐induced cardiomyopathy. Stem Cell Reports 7, 749–763. 27641650 10.1016/j.stemcr.2016.08.009 PMC5063626

[42] Morrison TJ , Jackson MV , Cunningham EK , Kissenpfennig A , Mcauley DF , O'Kane CM and Krasnodembskaya AD (2017) Mesenchymal stromal cells modulate macrophages in clinically relevant lung injury models by extracellular vesicle mitochondrial transfer. Am J Respir Crit Care Med 196, 1275–1286. 28598224 10.1164/rccm.201701-0170OC PMC5694830

[43] Collino F , Pomatto M , Bruno S , Lindoso RS , Tapparo M , Sicheng W , Quesenberry P and Camussi G (2017) Exosome and microvesicle‐enriched fractions isolated from mesenchymal stem cells by gradient separation showed different molecular signatures and functions on renal tubular epithelial cells. Stem Cell Rev Rep 13, 226. 28070858 10.1007/s12015-016-9713-1 PMC5380712

[44] Lai RC , Arslan F , Lee MM , Sze NSK , Choo A , Chen TS , Salto‐Tellez M , Timmers L , Lee CN , El Oakley RM *et al* (2010) Exosome secreted by MSC reduces myocardial ischemia/reperfusion injury. Stem Cell Res 4, 214–222. 20138817 10.1016/j.scr.2009.12.003

[45] Li QC , Liang Y and Su ZB (2019) Prophylactic treatment with MSC‐derived exosomes attenuates traumatic acute lung injury in rats. Am J Physiol Lung Cell Mol Physiol 316, L1107–L1117. 30892077 10.1152/ajplung.00391.2018

[46] Wu P , Zhang B , Shi H , Qian H and Xu W (2018) MSC‐exosome: a novel cell‐free therapy for cutaneous regeneration. Cytotherapy 20, 291–301. 29434006 10.1016/j.jcyt.2017.11.002

[47] Willis GR , Kourembanas S and Mitsialis SA (2017) Toward exosome‐based therapeutics: Isolation, heterogeneity, and fit‐for‐purpose potency. Front Cardiovasc Med 4, 63. 29062835 10.3389/fcvm.2017.00063 PMC5640880

[48] Yu B , Kim HW , Gong M , Wang J , Millard RW , Wang Y , Ashraf M and Xu M (2015) Exosomes secreted from GATA‐4 overexpressing mesenchymal stem cells serve as a reservoir of anti‐apoptotic microRNAs for cardioprotection. Int J Cardiol 182, 349–360. 25590961 10.1016/j.ijcard.2014.12.043 PMC4382384

[49] Lee C , Mitsialis SA , Aslam M , Vitali SH , Vergadi E , Konstantinou G , Sdrimas K , Fernandez‐Gonzalez A and Kourembanas S (2012) Exosomes mediate the cytoprotective action of mesenchymal stromal cells on hypoxia‐induced pulmonary hypertension. Circulation 126, 2601–2611. 23114789 10.1161/CIRCULATIONAHA.112.114173 PMC3979353

[50] Zhou Y , Xu H , Xu W , Wang B , Wu H , Tao Y , Zhang B , Wang M , Mao F , Yan Y *et al* (2013) Exosomes released by human umbilical cord mesenchymal stem cells protect against cisplatin‐induced renal oxidative stress and apoptosis in vivo and in vitro. Stem Cell Res Ther 4, 34–34. 23618405 10.1186/scrt194 PMC3707035

[51] Kim D , Nishida H , An SY , Shetty AK , Bartosh TJ and Prockop DJ (2016) Chromatographically isolated CD63+CD81+ extracellular vesicles from mesenchymal stromal cells rescue cognitive impairments after TBI. Proc Natl Acad Sci USA 113, 170–175. 26699510 10.1073/pnas.1522297113 PMC4711859

[52] Tan CY , Lai RC , Wong W , Dan YY , Lim SK and Ho HK (2014) Mesenchymal stem cell‐derived exosomes promote hepatic regeneration in drug‐induced liver injury models. Stem Cell Res Ther 5, 76. 24915963 10.1186/scrt465 PMC4229780

[53] Rager TM , Olson JK , Zhou Y , Wang Y and Besner GE (2016) Exosomes secreted from bone marrow‐derived mesenchymal stem cells protect the intestines from experimental necrotizing enterocolitis. J Pediatr Surg 51, 942–947. 27015901 10.1016/j.jpedsurg.2016.02.061 PMC4921266

[54] Fang S , Xu C , Zhang Y , Xue C , Yang C , Bi H , Qian X , Wu M , Ji K , Zhao Y *et al* (2016) Umbilical cord‐derived mesenchymal stem cell‐derived exosomal microRNAs suppress myofibroblast differentiation by inhibiting the transforming growth factor‐β/SMAD2 pathway during wound healing. Stem Cells Transl Med 5, 1425–1439. 27388239 10.5966/sctm.2015-0367 PMC5031180

[55] Bonafede R , Scambi I , Peroni D , Potrich V , Boschi F , Benati D , Bonetti B and Mariotti R (2016) Exosome derived from murine adipose‐derived stromal cells: neuroprotective effect on in vitro model of amyotrophic lateral sclerosis. Exp Cell Res 340, 150–158. 26708289 10.1016/j.yexcr.2015.12.009

[56] Wang X , Gu H , Qin D , Yang L , Huang W , Essandoh K , Wang Y , Caldwell CC , Peng T , Zingarelli B *et al* (2015) Exosomal miR‐223 contributes to mesenchymal stem cell‐elicited cardioprotection in polymicrobial sepsis. Sci Rep 5, 13721–13721. 26348153 10.1038/srep13721 PMC4562230

[57] Ono M , Kosaka N , Tominaga N , Yoshioka Y , Takeshita F , Takahashi R , Yoshida M , Tsuda H , Tamura K and Ochiya T (2014) Exosomes from bone marrow mesenchymal stem cells contain a microRNA that promotes dormancy in metastatic breast cancer cells. Sci Signal 7, ra63. 24985346 10.1126/scisignal.2005231

[58] Roccaro AM , Sacco A , Maiso P , Azab AK , Tai Y , Reagan M , Azab F , Flores LM , Campigotto F , Weller E *et al* (2013) BM mesenchymal stromal cell‐derived exosomes facilitate multiple myeloma progression. J Clin Invest 123, 1542–1555. 23454749 10.1172/JCI66517 PMC3613927

[59] Kordelas L , Rebmann V , Ludwig AK , Radtke S , Ruesing J , Doeppner TR , Epple M , Horn PA , Beelen DW and Giebel B (2014) MSC‐derived exosomes: a novel tool to treat therapy‐refractory graft‐versus‐host disease. Leukemia 28, 970. 24445866 10.1038/leu.2014.41

[60] Aslam M , Baveja R , Liang OD , Fernandez‐Gonzalez A , Lee C , Mitsialis SA and Kourembanas S (2009) Bone marrow stromal cells attenuate lung injury in a murine model of neonatal chronic lung disease. Am J Respir Crit Care Med 180, 1122–1130. 19713447 10.1164/rccm.200902-0242OC PMC2784417

[61] Batrakova EV and Kim MS (2016) Development and regulation of exosome‐based therapy products. Wiley Interdiscip Rev Nanomed Nanobiotechnol 8, 744–757. 26888041 10.1002/wnan.1395

[62] Linares R , Tan S , Gounou C , Arraud N and Brisson AR (2015) High‐speed centrifugation induces aggregation of extracellular vesicles. J Extracell Vesicles 4, 29509. 26700615 10.3402/jev.v4.29509 PMC4689953

[63] Witwer KW , Buzás EI , Bemis LT , Bora A , Lässer C , Lötvall J , Nolte‐'T Hoen EN , Piper MG , Sivaraman S , Skog J *et al* (2013) Standardization of sample collection, isolation and analysis methods in extracellular vesicle research. J Extracell Vesicles 2, 20360. 10.3402/jev.v2i0.20360 PMC3760646 24009894

[64] Li P , Mao Z , Peng Z , Zhou L , Chen Y , Huang P , Truica CI , Drabick JJ , El‐Deiry W , Dao M *et al* (2015) Acoustic separation of circulating tumor cells. Proc Natl Acad Sci USA 112, 4970–4975. 25848039 10.1073/pnas.1504484112 PMC4413297

[65] Hsu MF , Yu SH , Chuang SJ , Kuo TK , Singal PK , Huang CY , Kao CL and Kuo CH (2018) Can mesenchymal stem cell lysate reverse aging? Aging 10, 2900–2910. 30362957 10.18632/aging.101595 PMC6224235

[66] Albersen M , Fandel TM , Lin G , Wang G , Banie L , Lin C and Lue TF (2010) Injections of adipose tissue‐derived stem cells and stem cell lysate improve recovery of erectile function in a rat model of cavernous nerve injury. J Sex Med 7, 3331–3340. 20561166 10.1111/j.1743-6109.2010.01875.x PMC3885341

[67] Parekkadan B , van Poll D , Suganuma K , Carter EA , Berthiaume F , Tilles AW and Yarmush ML (2007) Mesenchymal stem cell‐derived molecules reverse fulminant hepatic failure (MSC‐CM reverses FHF). PLoS ONE 2, e941. 17895982 10.1371/journal.pone.0000941 PMC1978513

[68] Jeon D , Chu K , Lee S , Jung K , Ban J , Park D , Yoon H , Jung S , Yang H , Kim BS *et al* (2013) Neuroprotective effect of a cell‐free extract derived from human adipose stem cells in experimental stroke models. Neurobiol Dis 54, 414–420. 23376682 10.1016/j.nbd.2013.01.015

[69] Gauthaman K , Fong C , Arularasu S , Subramanian A , Biswas A , Choolani M and Bongso A (2013) Human Wharton's jelly stem cell conditioned medium and cell‐free lysate inhibit human osteosarcoma and mammary carcinoma cell growth in vitro and in xenograft mice. J Cell Biochem 114, 366–377. 22930595 10.1002/jcb.24367

[70] Chu K , Jeon D , Lee S , Jung K , Lee S and Roh J (2010) A cell‐free extract from human adipose stem cells protects mice against epilepsy. Annal Neurol 68, S38. 10.1111/j.1528-1167.2011.03182.x 21777228

[71] Khubutiya M , Temnov A , Vagabov V , Sklifas A , Rogov K and Zhgutov Y (2015) Effect of conditioned medium and bone marrow stem cell lysate on the course of acetaminophen‐induced liver failure. Bull Exp Biol Med 159, 118–123. 26033600 10.1007/s10517-015-2905-x

[72] Mishra P , Mishra P and Banerjee D (2012) Cell‐free derivatives from mesenchymal stem cells are effective in wound therapy. World J Stem Cells 4, 35–43. 22993660 10.4252/wjsc.v4.i5.35 PMC3443710

[73] Rajasingh J , Lambers E , Hamada H , Bord E , Thorne T , Goukassian I , Krishnamurthy P , Rosen KM , Ahluwalia D , Zhu Y *et al* (2008) Cell‐free embryonic stem cell extract‐mediated derivation of multi‐potent stem cells from NIH3T3 fibroblasts for functional and anatomical ischemic tissue repair. Cir Res 102, e107–e117. 10.1161/CIRCRESAHA.108.176115 PMC2435186 18483406

[74] Lin H , Fong C , Biswas A , Choolani M and Bongso A (2014) Human Wharton's jelly stem cells, its conditioned medium and cell‐free lysate inhibit the growth of human lymphoma cells. Stem Cell Rev Rep 10, 573–586. 24789672 10.1007/s12015-014-9514-3

[75] Lee C , Hsiao W and Lee O (2017) mesenchymal stem cell‐based therapies alleviate high‐fat diet‐induced obesity. Cytotherapy 19, S224.

[76] Shigemoto‐Kuroda T , Oh JY , Kim D , Jeong HJ , Park SY , Lee HJ , Park JW , Kim TW , An SY , Prockop DJ *et al* (2017) MSC‐derived extracellular vesicles attenuate immune responses in two autoimmune murine models: type 1 diabetes and uveoretinitis. Stem Cell Reports 8, 1214–1225. 28494937 10.1016/j.stemcr.2017.04.008 PMC5425726

[77] Riazifar M , Rezza Mohammadi M , Pone EJ , Yeri A , Lässer C , Segaliny AI , McIntyre LL , Vilas Shelke G , Hutchins E , Hamamoto A *et al* (2019) Stem cell‐derived exosomes as nanotherapeutics for autoimmune and neurodegenerative disorders. ACS Nano 13, 6670–6688. 31117376 10.1021/acsnano.9b01004 PMC6880946

[78] Hai B , Shigemoto‐Kuroda T , Zhao Q , Lee RH and Liu F (2018) Inhibitory effects of iPSC‐MSCs and their extracellular vesicles on the onset of sialadenitis in a mouse model of Sjögren's syndrome. Stem Cells Int 2018, 2092315. 29736173 10.1155/2018/2092315 PMC5875028

[79] Sharma J , Hampton JM , Valiente GR , Wada T , Steigelman H , Young MC , Spurbeck RR , Blazek AD , Bösh S , Jarjour WN *et al* (2017) Therapeutic development of mesenchymal stem cells or their extracellular vesicles to inhibit autoimmune‐mediated inflammatory processes in systemic lupus erythematosus. Front Immunol 8, 526. 28539924 10.3389/fimmu.2017.00526 PMC5423896

[80] Chen Z , Wang H , Xia Y , Yan F and Lu Y (2018) Therapeutic potential of mesenchymal cell‐derived miRNA‐150‐5p‐expressing exosomes in rheumatoid arthritis mediated by the modulation of MMP14 and VEGF. J Immunol 201, 2472–2482. 30224512 10.4049/jimmunol.1800304 PMC6176104

